# The Baltimore College of Dental Surgery

**Published:** 1868-11

**Authors:** 


### EDITORIAL DEPARTMENT.

*The Biltimore College of Dental Surgery.*—The Twenty Ninth Annual Session of the Baltimore College of Dental Surgery, commenced on the fifteenth of October, with the opening of the Infirmary and the delivery of preliminary lectures. The regular course of lectures commences on the second of November.

The friends of the Baltimore College will be pleased to learn that the number of matriculants at the time we go to press, is considerably greater than at the same period last year, and from the fact that the majority of second course students do not arrive until the regular lectures commence, being admitted as late as the 10th of November, there is good reason for believing that the class of 1868-69 will exceed that of the previous session.

The Faculty have lately imported from Paris a first class "Manikin" for demonstrations, which will prove a valuable addition to the large collection of Anatomical specimens belonging to this College. Few Medical Colleges are better supplied with anatomical and other specimens than the Baltimore College of Dental Surgery, and the lectures promise to become more and more interesting and instructive, as the Faculty are determined, in every way possible, to increase the facilities for obtaining a thorough dental education.

This College in spite of unbecoming and unfriendly criticism, has long ceased to be an experiment, and now enjoys an acknowledged place among the institutions of the world.

